# A Case of Recurrent Fixed Drug Eruption Secondary to Desloratadine

**DOI:** 10.7759/cureus.16762

**Published:** 2021-07-30

**Authors:** Eman Almukhadeb

**Affiliations:** 1 Dermatology, College of Medicine, King Saud University, Riyadh, SAU

**Keywords:** desloratadine, anti-histamine, fixed drug eruption, cutaneous drug eruption, delayed hypersensitivity

## Abstract

Desloratadine is a second-generation H1-type antihistamine that is widely prescribed for multiple indications including allergic rhinitis and urticaria. It is well-tolerated and is not known to cause cutaneous side effects including fixed drug eruption (FDE). In this report, a case of recurrent fixed drug eruption induced by desloratadine is reported.

## Introduction

Fixed drug eruption (FDE) is a type of cutaneous drug eruption that is characterized by single or multiple tenders or pruritic, well-demarcated, round-oval erythematous edematous plaques. It may develop a dusky violaceous hue or central bullae that later end up with erosion secondary to epidermal detachment [1]. It tends to develop few days to two weeks after the initial exposure to certain medication. It appears at the same site within 24 hours upon re-administration of the causative drug. Lesions can appear anywhere on the skin, as well as the mucous membranes, with common sites being the lips, face, hands, feet, and genitalia. When the lesions fade away, they often leave a residual post-inflammatory hyperpigmentation. Recurrence in the same location upon re-administration of a particular medication is the key to diagnosis. Provocation via patch testing in a previously involved site might be useful in determining the responsible drug [1,2].

The exact mechanism of FDE is unknown. Recent research suggests a cell-mediated process that initiates both the active and quiescent lesions. The process may involve an antibody-dependent, cell-mediated cytotoxic response. CD8+ effector/memory T cells play an important role in the reactivation of lesions with re-exposure to the offending drug [3,4].

The drugs most commonly associated with FDE include sulfonamides, tetracyclines, β-lactams, fluoroquinolones, macrolides, non-steroidal anti-inflammatory drugs (NSAIDs), acetaminophen, aspirin, barbiturates, dapsone, proton pump inhibitors, and azole antifungal drugs [5]. There are few reports of antihistamines as a cause of FDE [6-12].

Desloratadine is a selective H1 receptor antagonist and is the primary metabolite of loratadine. The literature review showed no reported cases of FDE secondary to desloratadine.

## Case presentation

**Figure 1:**
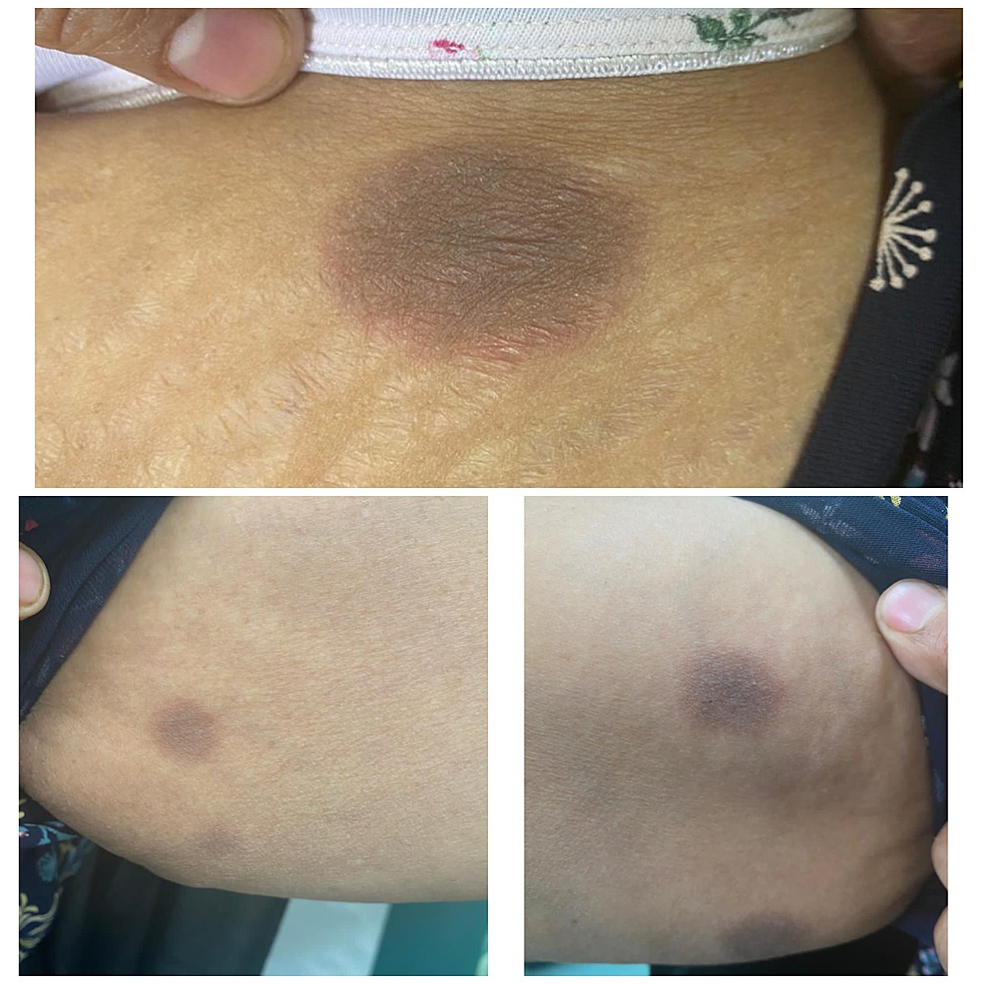
Well-defined, erythematous to violaceous patches and plaques over left arm, left thigh, and right arm.

A 59-year-old female was presented to the dermatology clinic at King Khaled University Hospital, Riyadh, with a one-year history of few slightly pruritic skin eruptions over arms and thighs. She is known to have diabetes mellitus controlled on metformin for the last nine years, hypertension controlled on nifedipine and irbesartan for the last seven years, and allergic rhinitis on mometasone furoate nasal inhaler and on-off desloratadine. She started to take desloratadine in February 2020 and she only takes it when her rhinitis symptoms flare up. The patient noticed that whenever she takes desloratadine she starts to develop these lesions at the same locations and they clear up in few days leaving post-inflammatory hyperpigmentation. On examination, she has five, well-defined, round, variable in size, erythematous to violaceous patches and plaques over the left thigh and both arms as shown in Figure 1. Mucous membranes were free.

**Figure 2:**
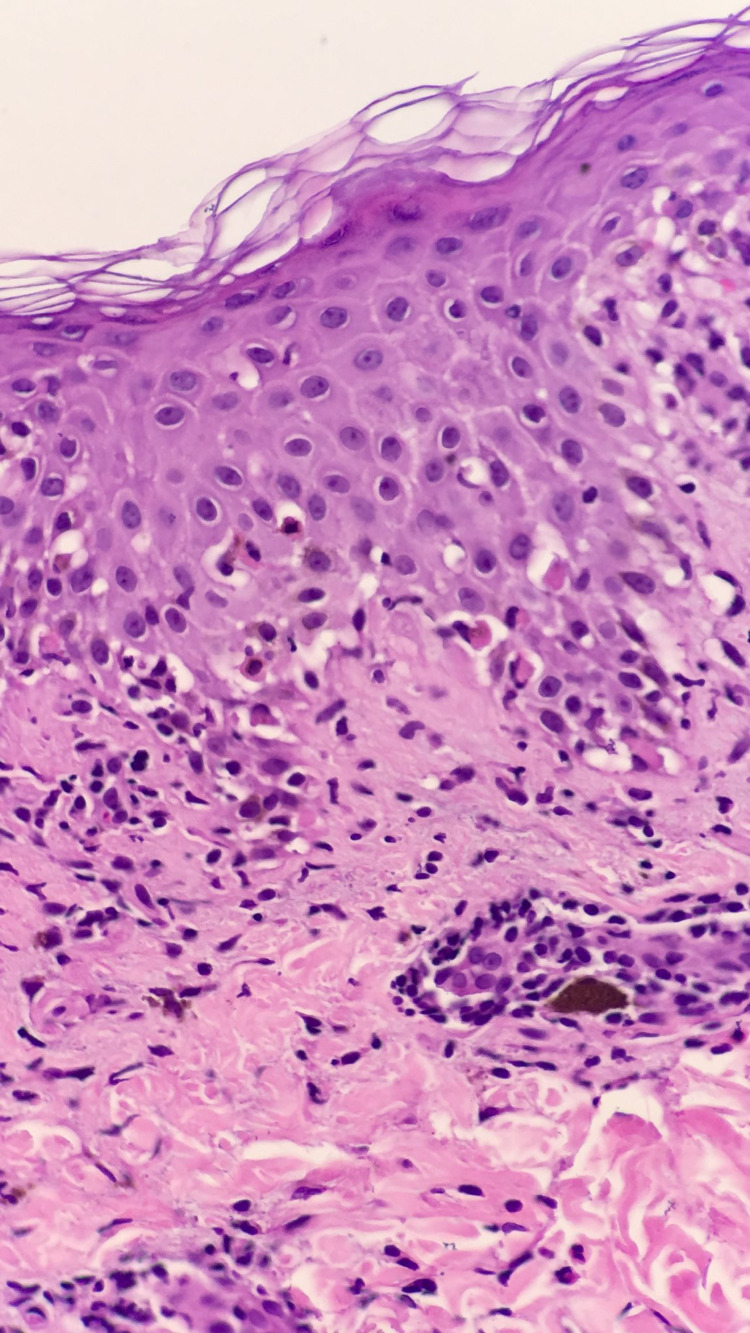
Interface dermatitis, scattered necrotic keratinocyte, dermal eosinophils, and melanophages.

A skin biopsy was obtained from the lesion at the right arm. Histologic examination revealed a normal basket weave stratum corneum, interface dermatitis, scattered necrotic keratinocyte, perivascular lymphocytes, dermal eosinophils, and melanophages consistent with the diagnosis of FDE, as shown in Figure 2.

**Figure 3:**
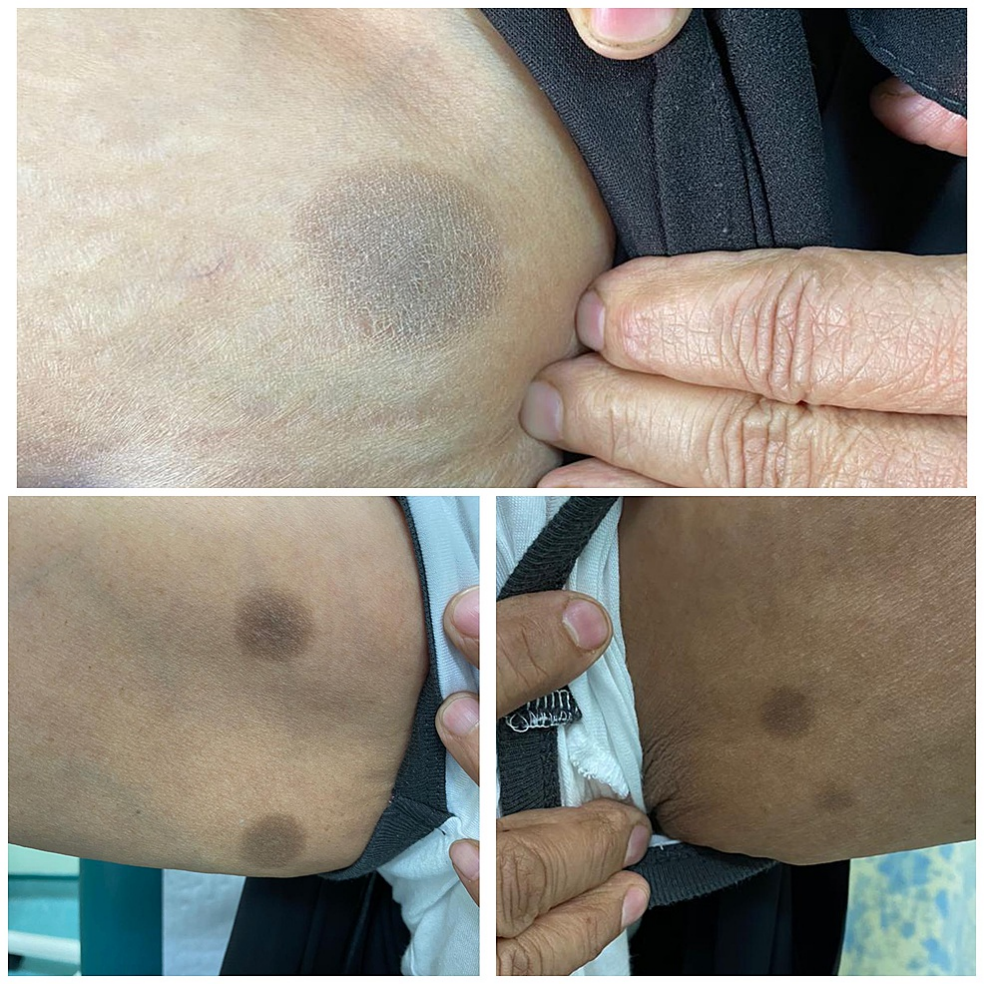
Two months after discontinuation of desloratadine with only post-inflammatory hyperpigmentation over left arm, right arm, left thigh.

The patient was referred to an allergy clinic for patch testing of the involved site which was provoked by the application of desloratadine. The patient was given topical mometasone furoate 0.1% ointment and advised to stop desloratadine. Two months later, her skin examination showed only post-inflammatory hyperpigmentation as shown in Figure 3, for which she was given hydroquinone 4% cream.

## Discussion

Fixed drug eruption is an uncommon type of cutaneous adverse drug eruption. It can be triggered by a large number of drugs including antibiotics, antiepileptics, nonsteroidal anti-inflammatory agents, phenothiazines, and other agents, and also certain foods such as cashews and licorice have also been reported as causative agents [5].

H1-antihistamines are widely used in clinical practice for the treatment of allergic disorders such as allergic rhinitis, urticaria, and other pruritic disorders. They have an excellent safety profile and are uncommonly associated with adverse cutaneous drug eruptions. FDE induced by first and second-generation H1-antihistamine is rare. There are few reports of FDE caused by hydroxyzine, cetirizine, diphenhydramine, dimenhydrinate, and loratadine [6-12].

A Medline search revealed no reported cases of FDE secondary to desloratadine and only two cases of FDE were induced by loratadine [8,9]. Since desloratadine is the active metabolite of loratadine, this study supports the previous two case reports of loratadine-induced FDE. Furthermore, it is expected that patients who developed FDE to desloratadine develop an FDE to loratadine as the first is the active metabolites of the second and it is recommended to use another antihistamine in such a patient.

In this case report, the diagnosis of FDE due to desloratadine was established based on recurrent episodes related to intermittent administration of desloratadine, i.e., positive oral rechallenge which is the gold standard for diagnosis, as well as a patch test, clinical and histologic findings.

The exact mechanism by which desloratadine induces FDE is unknown. It is thought that desloratadine act as a hapten that binds to basal keratinocytes, leading to an inflammatory response. This leads to the secretion of cytokines from keratinocytes including intercellular adhesion molecule-1 (ICAM1). ICAM1 helps T cells (CD4 and CD8) migrate to the insult site. CD8-T cells become reactivated after re-exposure to desloratadine and produce inflammatory cytokines, interferon-gamma, and tumor necrosis factor-alpha which leads to epidermal damage.

Oral rechallenge should not be routinely performed in FDE especially when positive patch testing brings additional evidence of the culpability of the drug. Oral rechallenge may involve major discomfort for the patient and may increase the occurrence of new lesions.

## Conclusions

In conclusion, it was assumed that this is the first report of the relationship between the oral ingestion of desloratadine and the production of a typically FDE. Since desloratadine is the active metabolite of loratadine, this patient might develop similar lesions if she takes loratadine in the future. So it is recommended to use different antihistamines other than loratadine and desloratadine.
